# Effects of virtual reality erotica on ejaculate quality of sperm donors: a balanced and randomized controlled cross-over within-subjects trial

**DOI:** 10.1186/s12958-022-01021-1

**Published:** 2022-10-11

**Authors:** Daniel Rosenkjær, Allan Pacey, Robert Montgomerie, Anne-Bine Skytte

**Affiliations:** 1 Cryos International Sperm and Egg bank, Vesterbro Torv 3, 8000 Aarhus C, Denmark; 2 Department of Oncology and Metabolism, The Jessop Wing, University of Sheffield, Level 4, Tree Root Walk, S10 2SF Sheffield, UK; 3 Department of Biology, Queen’s University, K7L 3N6 Kingston, ON Canada

**Keywords:** Ejaculate quality, Virtual reality, Male fertility, Sperm donation, Erotic stimulation

## Abstract

**Background:**

Previous research has shown that the type and duration of erotic material that men have access to during masturbation can influence semen parameters. To our knowledge, the use of virtual reality (VR) headsets to present erotica has not previously been studied. We reasoned that, because VR can provide a more immersive experience to the user, semen parameters of masturbatory ejaculates may be altered.

**Methods:**

This study had a balanced and randomized controlled cross-over within-subjects design. 504 ejaculates were collected from 63 sperm donors at 4 locations in Denmark. During masturbation each donor was instructed to observe erotic material either on a touch screen monitor or using a VR headset. The order of each pair of within-subject treatments was randomized by the throw of a dice. Anonymized data were analysed with linear mixed and piecewise structural equation models.

**Results:**

Both abstinence period and VR-use influenced the total number of motile spermatozoa ejaculated. For short abstinence periods, VR-use increased the number of motile sperm in the ejaculate. However, the difference between VR and non-VR ejaculates decreased as abstinence period increased such that there was no difference at the mean abstinence period of 58 h. For longer abstinence periods, total motile sperm counts were lower, on average, when men used VR compared to those that did not.

**Conclusion:**

The use of VR headsets to view erotica had a strong positive effect on the number of motile sperm in an ejaculate when the donor’s abstinence time was short (< 24 h). VR-use could improve the ejaculate quality of men who are asked to provide samples after a short period of abstinence, such as men in infertile partnerships producing samples for ART or cancer patients depositing sperm before treatment.

**Trial registration:**

Trial retrospectively registered on 13 July 2022 at ClinicalTrials.gov. Identifier: NCT05457764.

**Supplementary information:**

The online version contains supplementary material available at 10.1186/s12958-022-01021-1.

## Background

Human semen quality is affected by many variables such as season of the year [1], as well as donor age [2], BMI [3], occupation [4], cigarette smoking [5], stress [6] and time since last ejaculation [7]. The interplay of these variables leads to considerable variability in ejaculate quality within a single individual, posing a challenge for clinicians when attempting to assess male fertility [7, 8]. Of the many measures of semen quality, the total motile sperm count (TMSC) is the most closely associated with the probability of pregnancy following insemination [9].

Of the many potential influences on ejaculate quality, sexual stimulation is not well studied in humans. Some studies suggest that ejaculates obtained from heterosexual intercourse are of higher quality (by several measures) compared to ejaculates obtained by masturbation [10–12]. This could be attributed to the increased intensity and duration of sexual arousal achieved during intercourse [13].

In the present study we asked whether the use of virtual reality (VR) headsets to experience erotica has the potential to alter the ejaculate quality of sperm donors producing masturbatory ejaculates for clinical purposes (e.g., IVF). Here, a VR headset is a head-mounted device with stereoscopic display, stereo sound, and head-motion-tracking. The visual and auditory capabilities of VR create a virtual environment that is immersive and can invoke a unique sense of presence [14–16]. VR technology has found applications in numerous fields e.g., medicine, communication, engineering, psychology, and entertainment [17–19]. Men and women are increasingly engaging in emerging forms of sexual technology [20]. As a natural consequence, VR has found an application in erotica and studies have showed that VR induces higher degrees of sexual arousal than traditional two-dimensional erotica [21, 22]. Additionally, VR for the presentation of pornography made male participants feel elevated on multiple parameters compared to the two-dimensional control [23]. However, the study highlighted that the effects may have been caused by novelty of the medium and that they used single exposure, which may not reveal general effects. In the context of the abovementioned studies, we asked whether viewing erotic material by VR might serve to increase the level of sexual arousal during masturbation, either by relaxing the user or providing increased visual and auditory input in comparison to traditional erotica on paper or two-dimensional video.

## Materials and methods

### Data collection

The study design was within-subjects repeated measures, using balanced, randomized, controlled, cross-over sampling. Data was collected between 1 August and 25 November 2021 in the four largest cities in Denmark (Copenhagen, Aarhus, Odense, and Aalborg) from consenting sperm donors at Cryos International. A total of 63 ‘accepted’ donors (aged 19–44 years old and with a BMI between 17 and 35) participated in the study. The ejaculate quality criterion for becoming an ‘accepted’ donor at Cryos is to have a post thaw sperm concentration of ≥ 5 × 10^6^ per ml. Each participant donated 6–24 ejaculates, providing a total of 504 ejaculates for analysis. To be included in the study, donors had to donate at least three samples while using VR and three samples without, each on a different occasion. The VR setup involved a headset (Pico G2, Pico Interactive, San Fransisco, USA) offering a choice from 60 erotic videos each of 10–30 min duration. The men were allowed to watch any and as many of these video clips as they wished.

Sperm samples were donated in private rooms dedicated for the purpose and equipped with a touch screen showing erotic material. The men were asked to donate as usual and self-report the amount of time (in hours) since their last ejaculation (the abstinence period). Every other donation, donors rolled dice to determine whether a VR headset would be used; with the subsequent donation performed with the opposite condition. To maximize privacy and minimize stress on the donors, the amount of time the donors took to produce a sperm sample (donation period) was recorded as the number of seconds from closing the door to the private room to when it was opened again.

Each semen sample was weighed to determine ejaculate volume and allowed to liquify at room temperature for up to 1 h. After liquefaction, aliquots were loaded in duplicate onto Makler counting chambers (Sefi-Medical, Israel) and observed at 200x magnification using an Olympus CX41 microscope (Olympus, Japan). Measurements of sperm concentration, motility and motile sperm concentration were made using a MICROPTIC, S.L. (Barcelona, Spain) Computer Assisted Sperm Analysis system with at least 500 cells counted per analysis. No analysis of sperm morphology was performed.

### Statistical analyses

All 504 ejaculates were included in the analyses. Data was pseudoanonymized prior to analyses and no data was excluded (Supplementary Material Table S1). We used linear mixed models (LMMs) to assess the relationship between VR-use and ejaculate volume, donation period and total motile sperm count (TMSC) for each ejaculate sample, while controlling for several factors that might also influence ejaculates: (i) abstinence period, (ii) donor age, (iii) donor BMI, (iv) day of the year (season), and (v) location (donation site). In each model, we included a pseudoanonymized donor identity as a random effect to avoid pseudo-replication as each donor provided multiple samples. The interaction between VR-use and abstinence period was initially included in models as abstinence period is known to increase ejaculate size and quality [24, 25] though sometimes a decrease has been observed [26, 27]. We considered it to be at least plausible that the influence of VR might diminish as abstinence period increased and both ejaculate size and quality approached their maxima for each donor. We removed those interaction terms from models when p-values for the interactions was > 0.20.

To investigate the relative strength of plausible causal relationships among the variables in our LMMs, we used piecewise structural equation modeling [28]. To construct this model, we assumed that VR use might have a direct effect on ejaculate size, donation period and TMSC, and that ejaculate volume and donation period might have direct effects on TMSC (see Supplementary Material for model details).

We used R version 4.2.0 [29] for analyses, with packages *lmer* and *lmerTest* for LMMs. For interaction plots we used the *sjPlot* package. For piecewise structural equation modeling, we used the *pSEM* package with the *lme* function (*nlme* package) for mixed models and anonymized donor identity nested within location as random effects. To predict the magnitudes of effects from the full LMMs, we used the *ggpredict* function in the *ggeffects* package. All such effects are reported as mean [95%CL] calculated by setting the other predictors in the full models at their mean values. All R code and data are available on figshare (https://figshare.com/s/b0da6aaf446cc5b29062).

## Results

The use of VR to present erotica during sperm donation resulted in an increase in donation period, ejaculate volume, and TMSC (Table 1).

**Table 1:** Results of full LMMs to predict TMSC, donation period and ejaculate volume. In each model the response variables as well as abstinence period were log_10_-transformed to normalize distributions and residuals (Supplementary Material Figures S1, S2); all continuous predictors were standardized to facilitate comparison of effects on the same scale. NA indicates interaction terms not included in the full models because the p-values were > 0.20; bold text indicates statistical significance (P < 0.05). Each model is based on 504 samples from 63 donors

	Total Motile Sperm Count	Donation Period (min)	Ejaculate Volume (mL)
**Fixed effects**			
(Intercept)	**2.03 [1.94, 2.13] < 0.001**	**2.83 [2.77, 2.89] < 0.001**	**0.52 [0.46, 0.58] < 0.001**
VR used? [yes]	0.03 [–0.01, 0.07] 0.14	**0.06 [0.05, 0.08] < 0.001**	**0.02 [0.001, 0.03] 0.04**
Abstinence Period	**0.11 [ 0.07, 0.14] < 0.001**	0.00004 [–0.01, 0.01] > 0.99	**0.05 [0.04, 0.06] < 0.001**
VR-use x Abstinence Period	**–0.06 [–0.10, − 0.02] 0.005**	NA	NA
Age (years)	0.03 [–0.03, 0.08] 0.36	–0.02 [–0.06, 0.01] 0.24	0.015 [–0.021, 0.050] 0.42
BMI	–0.001 [–0.06, 0.05] 0.96	–0.008 [–0.04, 0.03] 0.66	–0.015 [–0.050, 0.020] 0.41
Day of the year	–0.003 [–0.03, 0.02] 0.83	–0.0009 [–0.01, 0.009] 0.85	0.003 [–0.006, 0.012] 0.45
Location [AAR]	**0.19 [0.03, 0.35] 0.02**	0.008 [–0.09, 0.11] 0.87	0.05 [–0.05, 0.15] 0.36
Location [CPH]	0.09 [–0.05, 0.23] 0.19	0.05 [–0.04, 0.13] 0.25	0.004 [–0.08, 0.09] 0.93
Location [ODE]	0.10 [–0.08, 0.27] 0.27	0.07 [–0.04, 0.18] 0.21	0.05 [–0.06, 0.16] 0.35
**Random effects**			
ICC	0.43	0.65	0.70
R^2^ marginal	0.16	0.08	0.14
R^2^ conditional	0.52	0.67	0.74

**Fig. 1:**
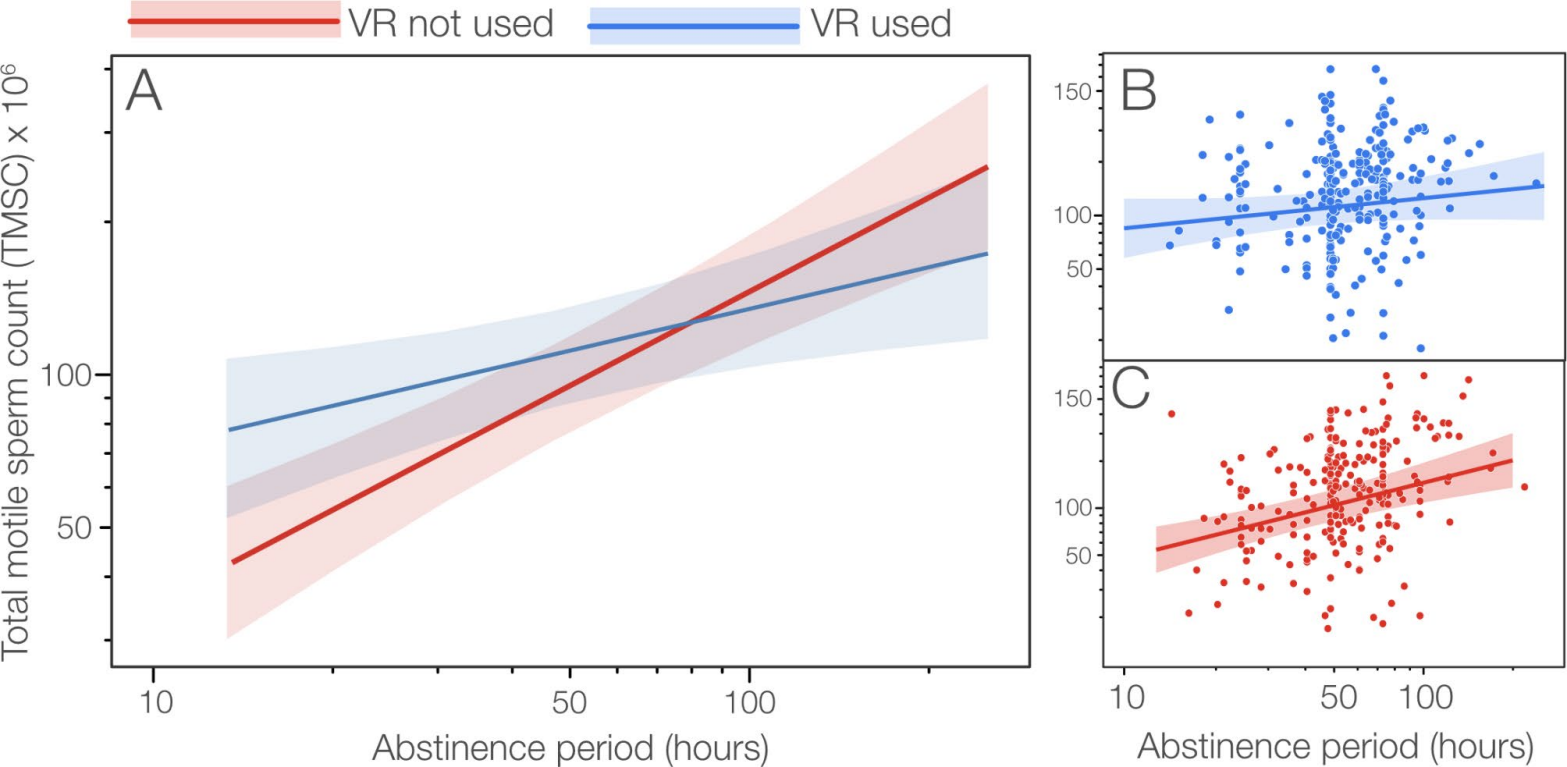
Effect of abstinence period on total motile sperm count (TMSC), with and without using VR. (A) interaction plot from the full model (Table 1) with 95% confidence limits on each predicted regression. (B, C) Separate predicted regressions from models for donors with and without VR, with symbols showing the raw data. Note the log scales on all graphs

The relationship between VR-use and TMSC, however, was confounded by the interaction between VR-use and abstinence period, such that the effect of abstinence period on TMSC was stronger (i.e., higher slope) when donors used VR (Fig. 1).

At the average period of abstinence (~ 58 h), there was no difference in TMSC between donors using or not using VR (Fig. 1 A). However, at shortest period of abstinence (14 h) donors who used VR during sperm donation produced, on average (90 [95%CL = 65, 124] x 10^6^ motile sperm) 33 million more motile sperm (TMSC) than donors who did not use VR (57 [41, 79] x 10^6^ motile sperm). At the longest period of abstinence (233 h), the pattern is reversed such that donors who used VR during sperm donation produced, on average (145 [95, 221] x 10^6^ motile sperm) 73 million fewer motile sperm (TMSC, Fig. 1B, C) than donors who did not use VR (218 [142, 334] x 10^6^ motile sperm).

**Fig. 2:**
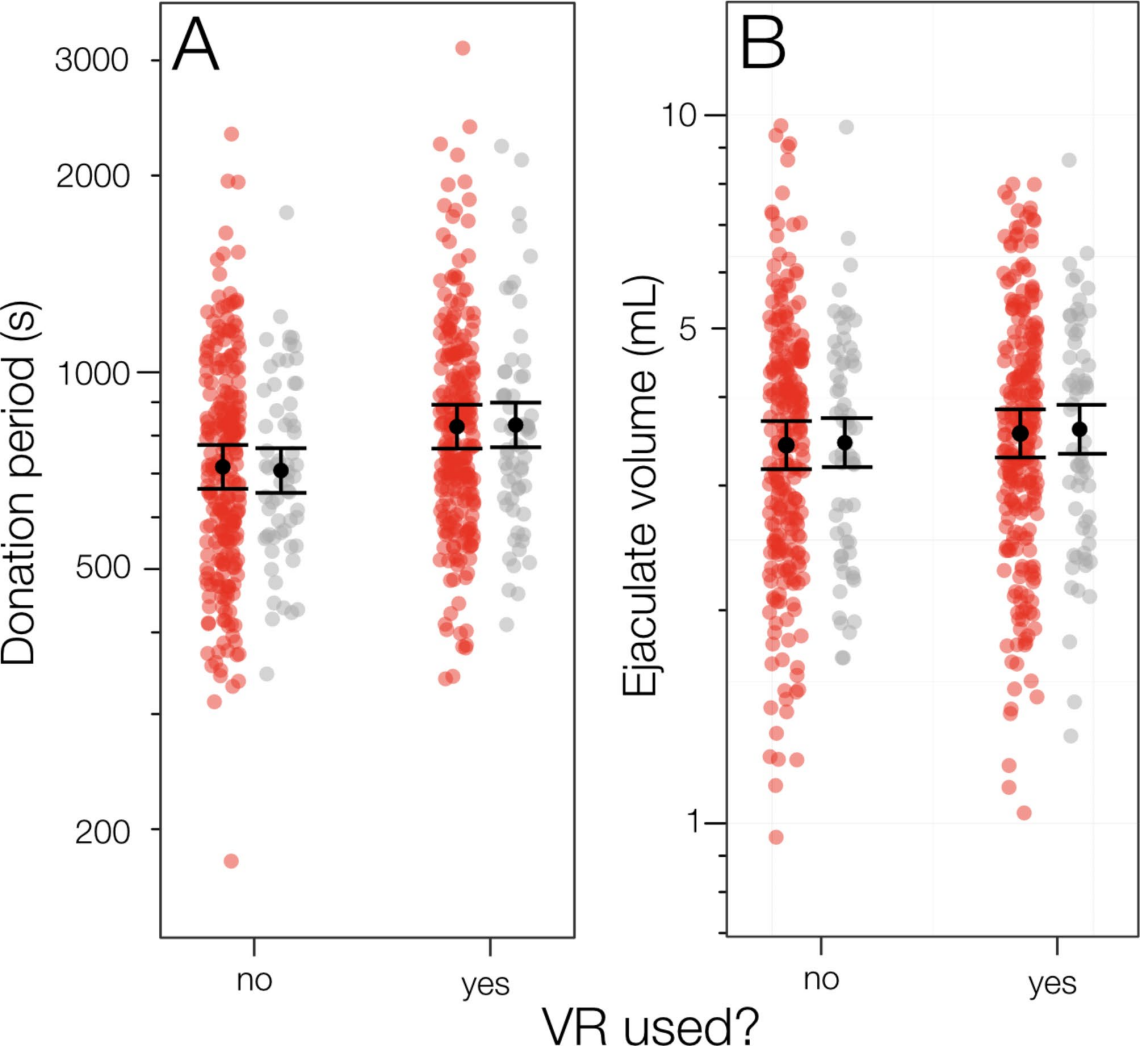
Effect of VR use on (A) the donation period and (B) ejaculate volume for each donor during each sperm donation (red, 252 donations in each category) and for the averages of all donations made by each donor (grey; 63 means in each category). Black symbols are means ± 95%CLs. Note the log scales on both graphs

The effects of VR-use on both ejaculate volume and donation period were not confounded by interaction between VR-use and the abstinence period but both effects were relatively small (Fig. 2).

Only VR-use and abstinence period were included in the best-fitting models to predict TMSC (Supplementary Material Table S2), and only location and donor age were included in the top models (∆AICc ≤ 2), given the available data. The average donation period for VR users (13.0 min [9.1, 14.8]) was only 1.8 min longer than for donors who did not use VR (11.2 [9.8, 12.9]), even though the difference is statistically significant (Table 1). Similarly, the average ejaculate volume for VR users (3.42 mL [2.87, 3.78] was only 0.13 mL larger than for donors who did not use VR (3.29 [3.20, 3.81]), even though that difference is also statistically significant (Table 1).

**Fig. 3:**
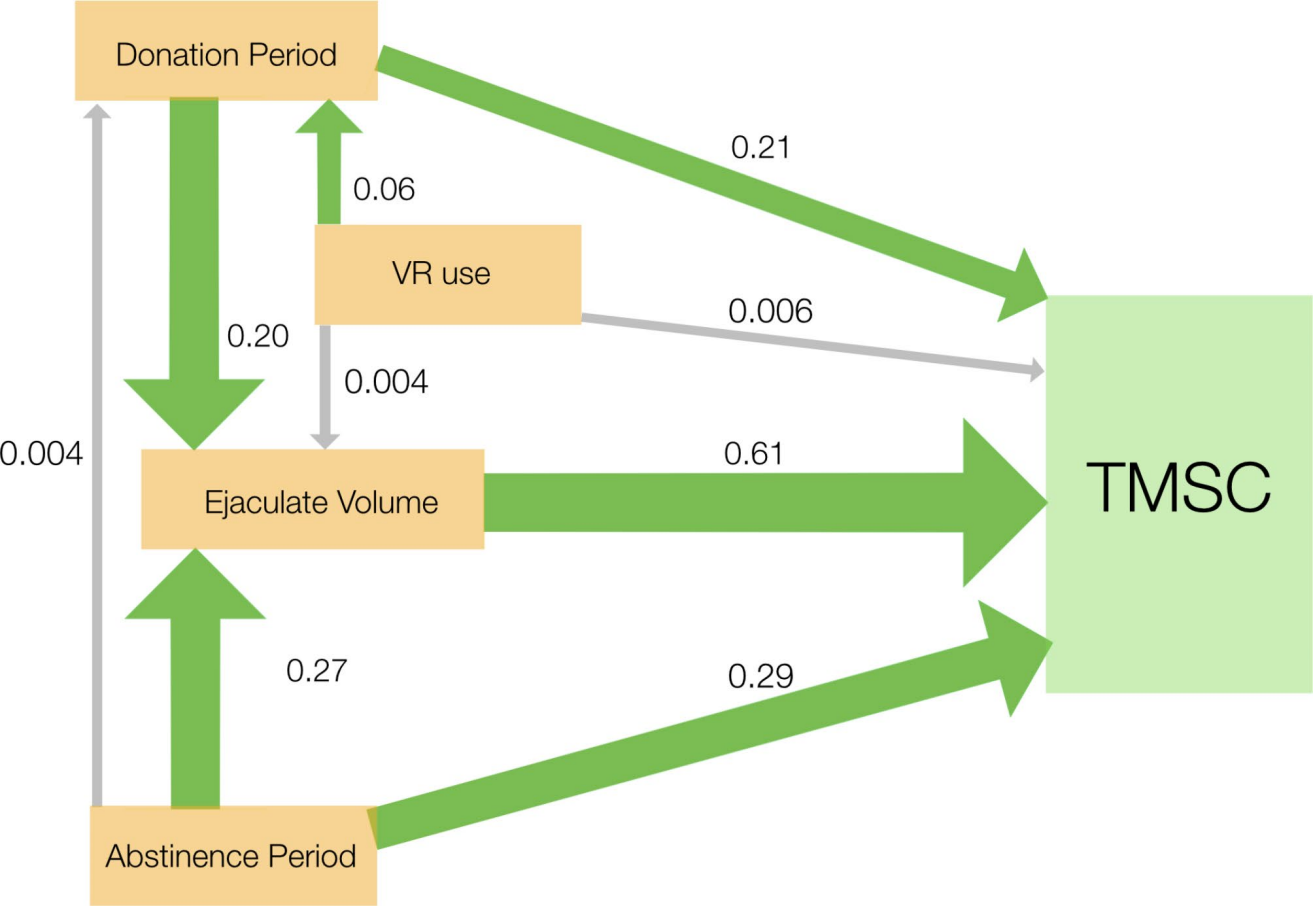
Path diagram from piecewise structural equation model showing the relative magnitude of effects; estimates on each arrow are standardized coefficients such that they are on the same scale and directly comparable. Green arrows are significant effects whereas grey arrows are not significant. The width of each arrow also indicates the magnitude of each effect. Arrows join variables that might plausibly influence one another and thus directly or indirectly have an effect on the total motile sperm count (TMSC).

The most plausible causal model derived from piecewise structural equation modeling (Fig. 3), shows that the direct effect of VR-use on TMSC was relatively small and not statistically significant. To reduce model complexity, we did not include donor age, donor BMI, or donation date in this model as none of those predictors were supported by any evidence in the full models (Table 1). Most of the effect of VR-use on TMSC arose indirectly through the effects of VR-use on the duration of the donation period and the effect of that donation period on ejaculate volume. The abstinence period had roughly equivalent effects on ejaculate volume and TMSC (Fig. 3) and very little effect on the donation period.

## Discussion

Our study has revealed a clear but complex effect of the presentation of erotic material via VR on total motile sperm count (TMSC) during sperm donation. This was largely through the effect of VR-use on the period of sperm donation (Figs. 1, 2 and 3). The donation period, in turn, had both direct and indirect (via its effect on ejaculate volume) effects on TMSC (Fig. 3). We do not know why the use of VR influences TMSC but we can suggest two possibilities worthy of further investigation.

First, it seems most likely to us that VR-use increased the erotic stimulation of donors as previously described [23] resulting in increased engagement with the erotic material and consequently longer periods of donation and higher sperm count. While donation period alone is a major factor affecting TMSC (Fig. 3), it seems unlikely that VR-use simply increased the donation period due to equipment setup and the duration of the erotic material watched. Second, it is possible that the delay in producing an ejaculate alone is responsible for increased TMSC. Thus, it seems most likely that the positive relationship between TMSC and donation period, whether or not the donor used VR, is due to the duration and quality of erotic stimulation experienced before ejaculation. These two, not mutually exclusive, possibilities could be investigated by recording the duration and nature of erotic stimulation experienced by a donor before ejaculation and correlating with the subjective experience of the donor.

Interestingly, VR-use did not have an appreciable direct effect on ejaculate volume (Fig. 3). This supports our finding that sperm count varies with the duration of the donation period which in turn influences ejaculate volume [30, 31]. The abstinence period also influenced sperm count directly, as well as indirectly through its effect on ejaculate volume (Fig. 3). Based on the standardized coefficients, ejaculate volume had 2–3 times as much influence on TMSC as the direct effects of the donation or abstinence periods. The interesting effect of VR-use on the relationship between TMSC and abstinence period is difficult to explain and deserves further study. At shorter abstinence periods (e.g., 14–24 h) VR-use increased average TMSC as expected. We also expected that the effect of VR-use on TMSC might decline with longer abstinence periods as donors approached their maximum ejaculate volumes and TMSCs. At long abstinence periods (e.g., 150–230 h), the higher TMSC when donors were not using VR compared to when they did is difficult to explain. Because each donor used VR as often as not, and treatments were randomized, this effect on TMSC at long periods of abstinence cannot be attributed to differences among donors.

While this study has revealed some interesting effects of VR-use on sperm counts, there is much unexplained variation (Fig. 1B C, 2) - the fixed effects in our statistical models explain only 16% of the variation in TMSC (Table 1), with 36% of the total variation due to within-donor variability and the remaining 48% unexplained. There are several plausible reasons for the unexplained variation, some of which could be addressed in future studies. First, abstinence period was self-reported and might well have been subject to error due to the donors dissembling or not understanding what that term means. Ejaculations can also be cryptic (nocturnal emissions) resulting in an overestimation of the period of ‘abstinence’ that might influence sperm count. Second, while donation period was accurately recorded, it included unknown periods of time (i) preparing to begin engaging with the erotic material, (ii) choosing which erotic material to access, (iii) switching between erotic stimuli, and (iv) preparing to leave the donation room after ejaculation. Any of these factors could lead to variation in the donation period that has no effect on TMSC. Donors might also find some types of erotic material more stimulating than others and might have switched to different stimulants during the donation period, lengthening that period and increasing the delays to ejaculation. Third, additional parameters which have showed to affect ejaculate quality (e.g., occupation, smoking and stress) were not recorded. Finally, we did not record the activity of donors during the abstinence period and these activities might have influenced ejaculate volume and TMSC (e.g., partying versus studying).

This study is the first to look at the use of VR to display erotica in the context of ejaculate quality. The within-subjects study design provides strong statistical power as the subjects were their own control. Furthermore, donors each provided a minimum of three ejaculate samples for each treatment condition over the course of several months, minimizing seasonal effects on sperm quality. The ejaculates were from ‘accepted’ donors, which are a selected population of men known to produce high quality ejaculates. This means that we may not generalize the findings to other groups such as infertile partnerships or men seeking to cryopreserve their sperm for other reasons (e.g., prior to cancer treatment).

## Conclusion

The results of this study show a clear advantage of VR-use during sperm collection in a clinical setting when the donor’s period of abstinence was short (< 24 h). Thus, we found an increased sperm count of almost 50%, on average, at the shortest abstinence period that we studied (14 h) and would expect an even larger effect at even shorter periods. This advantage will need to be evaluated in light of the increased cost of using VR equipment, but we expect that the long-term efficiencies will be substantial. We cannot, however, recommend the use of VR when the abstinence period is longer than 30 h as that would provide no advantage and potentially some reduction in sperm count when it exceeds 100 h.

These promising findings suggest that further research on the use of VR to display erotica during sperm donation is warranted, especially to determine whether our results are generalizable to other populations. Further work should also test different erotic stimuli available through VR, as well as the various potential sources of unexplained variation in sperm count that we have described.

## Electronic supplementary material

Below is the link to the electronic supplementary material.

Supplementary Material 1

## Data Availability

The data analysed for this article and the R code used for analyses are publicly available at https://figshare.com/s/b0da6aaf446cc5b29062.
