# Supplementary material for: Effects of virtual reality erotica on ejaculate quality of sperm donors: a balanced and randomized controlled cross-over within-subjects trial
Source: Reprod Biol Endocrinol. 2022 Oct 11;20:149. doi: 10.1186/s12958-022-01021-1 (PMC9552463; doi:10.1186/s12958-022-01021-1)
Supplement: Supplementary file 1 — Supplementary Material 1 [file 12958_2022_1021_MOESM1_ESM.pdf]

SUPPLEMENTARY MATERIAL

This document provides additional statistical details and graphs for the paper:

Rosenkjær D, Pacey A, Montgomerie R, Skytte A-B. 2022. Erotica by virtual reality improves ejaculate quality of sperm donors. Manuscript submitted.

1 Descriptive Statistics

Descriptive statistics for the VR and non-VR treatments are listed in Table S1 where all of the raw data are analyzed even though there were multiple donations from each donor. The distributions of raw data for some key variables are shown in Figure S1, before and after log10-transformations to help normalize the distributions. While the Shapiro-Wilk normality tests indicated that the distributions of the log-transformed variables are significantly different from normal (Gaussian) comparing the distributions on the graphs shows that the log-transformed variables are a much better fit to normality and certainly within the acceptable limits for linear mixed models.

Table S1 Descriptive statistics of raw data when using VR (upper table) and not using VR (lower table).

VR: y

	vars	n	mean	sd	median	trimmed	mad	min	max	range	skew	kurtosis	se
Donor	1	252	30.94	18.28	31.00	30.91	23.72	1.00	63.00	62.00	-0.02	-1.20	1.15
Age	2	252	26.63	5.98	25.00	25.48	2.97	19.00	44.00	25.00	1.67	2.06	0.38
BMI	3	252	23.79	2.85	23.40	23.54	2.37	17.20	35.30	18.10	1.12	2.50	0.18
EjacNo	4	252	81.10	62.91	65.50	71.71	45.22	2.00	252.00	250.00	1.21	0.89	3.96
ProdDate*	5	252	35.98	15.29	36.00	36.06	18.53	4.00	67.00	63.00	-0.04	-0.93	0.96
DeptCode*	6	252	2.22	1.15	2.00	2.15	1.48	1.00	4.00	3.00	0.22	-1.47	0.07
EjacVOL	7	252	3.80	1.36	3.70	3.72	1.48	1.00	7.70	6.70	0.47	-0.42	0.09
PreA	8	252	9.34	10.17	6.00	7.67	5.93	0.00	60.00	60.00	1.89	4.70	0.64
PreB	9	252	29.20	20.04	23.00	26.58	16.31	1.00	107.00	106.00	1.20	1.25	1.26
PreC	10	252	36.79	27.81	30.00	32.39	17.79	1.00	209.00	208.00	2.38	8.45	1.75
PreDens	11	252	93.37	50.93	80.00	87.44	42.25	8.00	325.00	317.00	1.41	2.94	3.21
AbstTime	12	252	58.51	26.28	51.00	56.16	19.27	14.00	233.00	219.00	1.91	8.36	1.66
DonTime	13	252	875.39	354.12	798.50	824.12	222.39	385.00	2977.00	2592.00	2.16	7.05	22.31
VR*	14	252	2.00	0.00	2.00	2.00	0.00	2.00	2.00	0.00	NaN	NaN	0.00
DOY	15	252	272.22	18.48	272.00	272.11	21.50	232.00	321.00	89.00	0.06	-0.67	1.16

VR: n

	vars	n	mean	sd	median	trimmed	mad	min	max	range	skew	kurtosis	se
Donor	1	252	30.94	18.28	31.00	30.91	23.72	1.00	63.00	62.00	-0.02	-1.20	1.15
Age	2	252	26.63	5.98	25.00	25.48	2.97	19.00	44.00	25.00	1.67	2.06	0.38
BMI	3	252	23.79	2.85	23.40	23.54	2.37	17.20	35.30	18.10	1.12	2.50	0.18
EjacNo	4	252	80.92	62.82	66.00	71.45	45.96	1.00	253.00	252.00	1.23	0.92	3.96
ProdDate*	5	252	34.72	15.27	34.50	35.09	17.05	1.00	68.00	67.00	-0.15	-0.76	0.96
DeptCode*	6	252	2.22	1.15	2.00	2.15	1.48	1.00	4.00	3.00	0.22	-1.47	0.07
EjacVOL	7	252	3.68	1.45	3.45	3.56	1.41	1.20	9.60	8.40	0.98	1.58	0.09
PreA	8	252	9.82	12.64	5.00	7.39	5.93	0.00	92.00	92.00	2.87	11.96	0.80
PreB	9	252	28.18	22.49	22.00	24.87	14.83	1.00	190.00	189.00	2.48	11.18	1.42
PreC	10	252	34.51	31.31	26.50	29.07	17.05	0.00	208.00	208.00	2.95	11.36	1.97
PreDens	11	252	91.25	58.22	79.00	82.65	39.29	7.00	361.00	354.00	1.91	4.85	3.67
AbstTime	12	252	58.19	27.30	50.50	55.24	21.50	14.00	216.00	202.00	1.71	5.57	1.72
DonTime	13	252	758.51	280.89	717.50	731.05	263.16	207.00	2201.00	1994.00	1.32	3.30	17.69

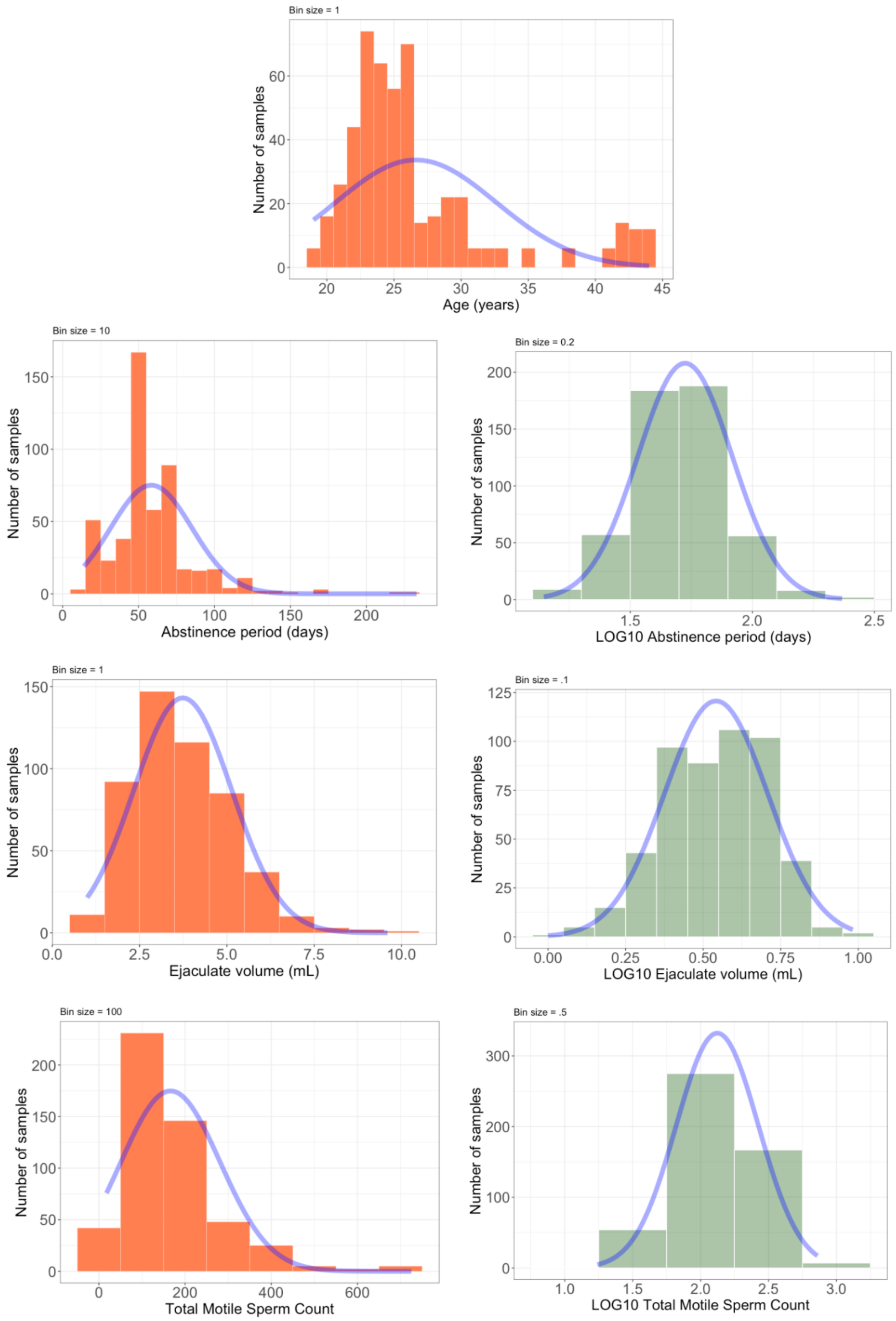

Figure S1 Frequency distributions of raw (orange bars,) and log10-transformed data on donor age, abstinence period, ejaculate volume and TMSC. Blue lines are normal curves.

2 Linear Mixed Models (LMM)

To to evaluate the factors that might influence TMSC, donation period, and ejaculate volume we constructed linear mixed models with VR use (yes/no) and abstinence period and their interaction, as well as donor age, BMI, day of the year and donation location as predictors, with donor identity (anonymized) as a random effect. The full models are presented in Table 1 of the paper, with diagnostic tests in Figure S2.

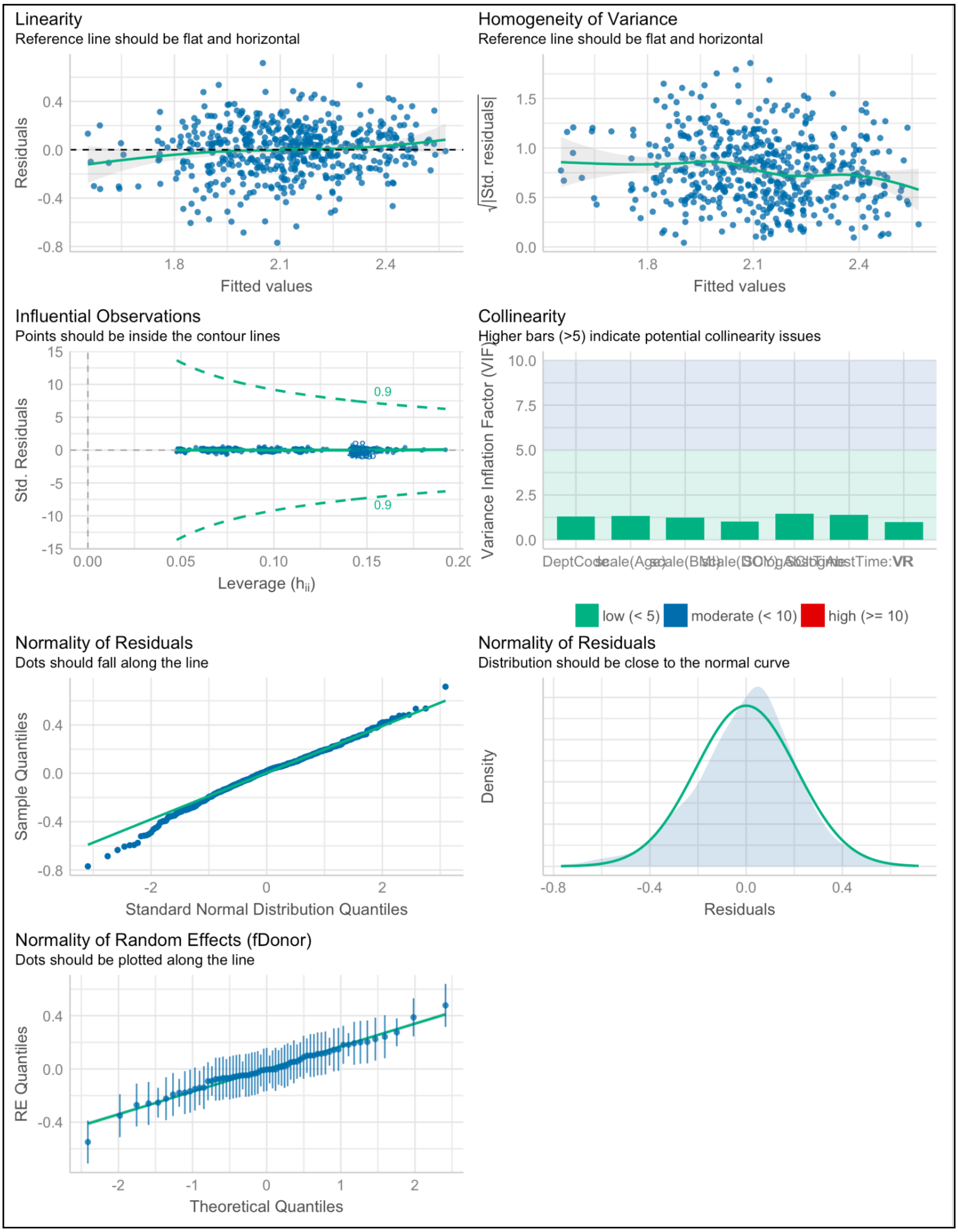

Figure S2 Diagnostic plots to test assumptions for full model to predict TMSC in Table 1

We used the *dredge* function in the *MuMIn* package in R to compare and evaluate all of the submodels based on those predictors. The top models from that analysis are shown in Table S2.

We designated top models to be those with ΔAICc<2 as these are considered to be statistically equivalent, given the data. The best-fitting model is the first model in each table,

Table S2 Top models (AICc ≤2 to predict (A) TMSC, (B) donation period and (C) ejaculate volume. In these tables scl = scaled, DOY = day of the year, SCA = scaled log10-abstinence period, DpC = location, SCA:VR = interaction between VR use and abstinence period. See Arnold (2010) on informative parameters.

Table S2A Top models to predict TMSC. Note that models 115 and 121 do not improve the loglikelihood, suggesting that the parameters included in those models are not informative.

Model selection table

	(Int)	DpC	scl(Age)	scl(BMI)	scl(DOY)	SCA	VR	SCA:VR	df	logLik	AICc	delta	weight
113	2.112					0.11490	+	+	6	-16.642	45.5	0.00	0.186
114	2.032	+				0.10780	+	+	9	-13.867	46.1	0.64	0.135
115	2.111		0.01899			0.11390	+	+	7	-16.349	46.9	1.47	0.089
116	2.033	+	0.02579			0.10660	+	+	10	-13.336	47.1	1.67	0.081
121	2.112				-0.002605	0.11490	+	+	7	-16.618	47.5	2.01	0.068

Table S2B Top models to predict donation period. Note that models 35 and 37 do not improve the loglikelihood, suggesting that the parameters included in those models are not informative.

Model selection table

	(Int)	DpC	scl(Age)	scl(BMI)	scl(DOY)	SCA	VR	df	logLik	AICc	delta	weight
33	0						+	4	393.818	-779.6	0.00	0.158
37	0			-0.12600			+	5	394.614	-779.1	0.45	0.126
35	0		-0.11760				+	5	394.553	-779.0	0.57	0.119
39	0		-0.08540	-0.09542			+	6	394.962	-777.8	1.80	0.064
49	0					0.0047600	+	5	393.825	-777.5	2.03	0.057
41	0				0.003544		+	5	393.824	-777.5	2.03	0.057

Table S2C Top models to predict ejaculate volume

Model selection table

	(Int)	DpC	scl(Age)	scl(BMI)	scl(DOY)	SCA	SCD	VR	SCD:VR	df	logLik	AICc	delta	weight
49	0					0.3127	0.1952			5	438.830	-867.5	0.00	0.167
57	0				0.02008	0.3126	0.1950			6	439.102	-866.0	1.51	0.079
241	0					0.3136	0.1574	+	+	7	440.042	-865.9	1.68	0.072
51	0		0.05549			0.3116	0.1961			6	439.005	-865.8	1.70	0.072
53	0			-5.272e-02		0.3131	0.1942			6	438.979	-865.8	1.75	0.070
113	0					0.3123	0.1892	+		6	438.958	-865.7	1.79	0.068
55	0		0.08311	-8.232e-02		0.3118	0.1950			7	439.327	-864.4	3.11	0.035
[illegible]	[illegible]		[illegible]		[illegible]	[illegible]	[illegible]			[illegible]	[illegible]	[illegible]	[illegible]	[illegible]

3 Piecewise Structural Equation Model

We used the following LMMs to inform the structural equation model (see R script 'Rhumans.Rmd' for full model details):

- logTMScount ~ logAbstTime +VRdum +logDonTime +logEjacVOL, random = ~1|DeptCode/fDonor, method = "ML"
- logDonTime ~ logAbstTime +VRdum, random = ~1|DeptCode/fDonor, method = "ML"
- logEjacVOL ~ logAbstTime +VRdum + logDonTime, random = ~1|DeptCode/fDonor, method = "ML"

In each LMM, donor identity (fDonor) is nested within location (DeptCode) and VR use (VRdum) is entered as a dummy variable (0,1) as required by the *lme* function that we used. To simplify model structures we did not include donor age, donor BMI, or day of the year in these models as none of those variables were included in any of the best-fitting models (Table S2).

REFERENCES

Arnold, T. W. (2010). Uninformative parameters and model selection using akaike's information criterion. The Journal of wildlife management 74:1175–1178.
